# Esketamine-based PCIA combined with intercostal nerve block for acute pain after lobectomy: a randomized controlled trial

**DOI:** 10.3389/fphar.2026.1746121

**Published:** 2026-03-04

**Authors:** Meiyan Zhou, Yu Qi, Fan Zhou, Hui Wu, Jiao Chen, Long Wang, Liwei Wang

**Affiliations:** 1 Xuzhou Clinical College, Xuzhou Medical University, Xuzhou, Jiangsu, China; 2 Department of Anesthesiology, Xuzhou Central Hospital, Xuzhou, Jiangsu, China; 3 Department of Anesthesiology, Xuzhou Central Hospital, Southeast University, Xuzhou, Jiangsu, China; 4 Jiangsu Province Key Laboratory of Anesthesiology, Xuzhou Medical University, Xuzhou, Jiangsu, China; 5 College of Anesthesiology, Xuzhou Medical University, Xuzhou, Jiangsu, China

**Keywords:** esketamine, intercostal nerve block, lobectomy, opioid-sparing analgesia, postoperative pain

## Abstract

**Objective:**

To evaluate outcomes of different doses of esketamine in postoperative patient-controlled intravenous analgesia (PCIA) combined with preoperative intercostal nerve block (ICNB) analgesia protocol for acute postoperative pain (APP) relief in patients undergoing thoracoscopic lobectomy.

**Materials and methods:**

A total of 360 patients scheduled for thoracoscopic lobectomy at Xuzhou Central Hospital between October 2021 and July 2023 were enrolled and randomly assigned into three groups using the random envelope method. Before anesthesia induction, all patients received ICNB, followed by general anesthesia. Group C received PCIA using sufentanil at 0.03 μg/kg/h, Group K1 received a low dose of esketamine at 0.015 mg/kg/h in PCIA, and Group K2 received a moderate dose of esketamine at 0.03 mg/kg/h in PCIA. Numerical Rating Scale (NRS) pain scores were recorded at 2, 4, 24, 48, and 72 h postoperatively. The incidence of APP within 72 h post-surgery, the requirement for rescue analgesia and the occurrence of adverse reactions, were assessed and recorded for the three groups.

**Results:**

There were significantly lower NRS scores in Group K2 at 2, 4, 24, 48, and 72 h postoperatively, compared to Groups C and K1 (P < 0.01). However, there were no statistically significant differences in NRS scores between Group C and Group K1 (P > 0.05). The incidence of APP within 72 h postoperatively was significantly lower in Group K2, compared to Group C and Group K1 (P < 0.05). Additionally, Group C exhibited a significantly higher the incidence of postoperative nausea and vomiting (PONV) than the other two groups (P < 0.05). Group K2 demonstrated superior postoperative analgesic efficacy, including reduced rescue analgesia needs and lower opioid consumption, without affecting hospital stay length compared to Groups C and K1.

**Conclusion:**

A dose of 0.03 mg/kg/h esketamine in PCIA combined with preoperative ICNB significantly alleviates APP in patients undergoing thoracoscopic lobectomy, reducing resting pain scores by approximately 30% at 24 h compared to sufentanil-based analgesia.

**Clinical Trial Registration:**

https://www.chictr.org.cn/, Identifier ChiCTR2100051000.

## 1 Introduction

Thoracic surgery, including lobectomy, is associated with severe APP due to various factors such as surgical trauma, drainage tube placement, intercostal nerve injury or compression, pleural damage, and postoperative coughing (Jin et al., 2025). Although video-assisted thoracoscopic surgery (VATS) has largely replaced open thoracotomy as a less invasive alternative, studies report that the incidence of APP following VATS remains as high as 40% (Bendixen et al., 2016; Kawaguchi et al., 2024). Additionally, inadequate postoperative pain management significantly increases the risk of developing chronic postoperative pain, with reported incidences ranging from 22% to 63% (Marshall and McLaughlin, 2020; Moorthy et al., 2023). Therefore, improving postoperative analgesic regimens and enhancing recovery quality for patients remain critical challenges for anesthesiologists.

Currently, opioids remain important and widely used agents for postoperative pain management. However, due to their various adverse effects such as nausea, vomiting, ileus, delirium, delirium, and hyperalgesia (Barakat et al., 2024), opioid-sparing strategies have gained increasing attention (Gilson and Khandhar, 2023). These strategies involve combining multimodal analgesic techniques and medications (e.g., perioperative nerve blocks) to achieve the lowest effective opioid dose over the shortest possible duration, which can effectively facilitate rapid postoperative recovery and reduce opioid dependence (Zhang et al., 2025). In thoracic surgery, preoperative regional nerve blocks, such as ICNB, are a key component of multimodal analgesia. They effectively manage somatic pain in the surgical area and are recognized by the European Society of Thoracic Surgeons (ESTS) as part of the enhanced recovery after surgery (ERAS) protocols for thoracic surgery (Spaans et al., 2025; Sertcakacilar et al., 2022).

In this context, esketamine, a non-opioid analgesic with a unique mechanism of action, offers a new possibility for developing opioid-sparing PCIA regimens. As the S-enantiomer of ketamine and a potent N-methyl-D-aspartate (NMDA) receptor antagonist, esketamine inhibits central sensitization, which may help prevent or delay the transition from acute to chronic pain. (Mizobuchi et al., 2022). Furthermore, studies suggest that subanesthetic doses can improve postoperative mood and sleep quality (Chen et al., 2025), aligning well with the goals of ERAS. Unlike traditional opioids, esketamine lacks direct depressive effects on the respiratory center, significantly reducing the risk of respiratory depression (Zhong et al., 2023). This safety profile makes it particularly suitable for patients undergoing pulmonary surgery.

However, current evidence is primarily limited to the use of esketamine as an adjunct to opioid analgesia (Li et al., 2024; Zhang et al., 2023). High-quality evidence is lacking on whether esketamine can completely replace opioids in a PCIA regimen—particularly in combination with a preoperative ICNB—with respect to the required dose, analgesic effectiveness, and safety profile. To this end, we conducted a prospective, double-blind, randomized controlled trial. The objectives of this trial were to: (Jin et al., 2025): assess the feasibility of an esketamine-based patient-controlled intravenous analgesia regimen for video-assisted thoracoscopic lobectomy; (Bendixen et al., 2016); determine the optimal dose of esketamine when used in combination with nerve blockade; and (Kawaguchi et al., 2024) determine the minimum effective dose of esketamine when used in combination with nerve blockade.

## 2 Methods

### 2.1 Study design

This was a single-center, prospective, randomized controlled trial approved by the Ethics Committee of Xuzhou Central Hospital (Approval No.: XZXY-LJ-20210331-044) and registered with the Chinese Clinical Trial Registry on 10/09/2021 (Registration No.: ChiCTR2100051000). The registration details can be accessed at https://www.chictr.org.cn/searchproj.html?regno=ChiCTR2100051000. Informed consents were obtained from all patients and the study adheres to the CONSORT guidelines for reporting randomized clinical trials. We confirm that all experiments were performed in accordance with relevant guidelines and regulations.

### 2.2 Inclusion and exclusion criteria

A total of 734 patients scheduled for thoracoscopic radical lung cancer surgery at Xuzhou Central Hospital between October 2021 and July 2023 were selected for recruitment. Inclusion criteria were as follows: elective and non-emergency patients; patients scheduled for thoracoscopic lobectomy; patients attaining American Society of Anesthesiologists (ASA) classification I-III; conscious and alert patients; patients aged 30–75 years; and informed consent obtained from the patient. Exclusion criteria were as follows: presence of rib fractures, infection at the nerve block puncture site, and a history of chest wall surgery; contraindications to surgery; contraindications to the study medications; severe cardiac, pulmonary, hepatic, renal, or coagulation disorders; and a history of psychiatric illness. Additionally, dropout criteria encompassed patients experiencing acute breakthrough pain postoperatively or those requiring a second thoracotomy. Patients who experienced acute breakthrough pain or required a second thoracotomy were recorded as discontinuing the study intervention; however, they were not excluded from the efficacy analysis. All data from these patients were included in the statistical analysis according to the intention-to-treat principle.

### 2.3 Randomization and blinding

This study used a random envelope method to ensure allocation concealment. Participants were randomly assigned into one of the three groups in a 1:1:1 ratio in a double-blind design. Opaque envelopes, externally labeled with enrollment numbers and internally marked with group assignment, were used to maintain blinding. A third party, independent of the study team, opened the envelopes sequentially in accordance with the order of the patient visits and implemented the intervention specified inside the envelope. Both the patients and researchers remained blinded to group assignment and interventions throughout the study. Prior to enrollment, all patients provided written informed consent. The consent form detailed the potential analgesic interventions (sufentanil-based or esketamine-based PCIA combined with nerve block) and the randomized, double-blind study design, ensuring participants remained blinded to their group allocation. A total of 360 patients were recruited and evenly assigned into the three groups: Group C, Group K1, and Group K2, with 120 patients in each group.

### 2.4 Anesthesia and monitoring

Anesthesia induction was performed through intravenous injection of sufentanil (0.4–0.6 μg/kg), propofol (2–3 mg/kg), and cisatracurium besylate (0.2 mg/kg). Total intravenous anesthesia (TIVA) was achieved using a combination of propofol (4–10 mg/kg/h) and remifentanil (0.1–1.0 μg/kg/min). Continuous infusion of cisatracurium besylate (0.15 mg/kg/h) was maintained, with End-Tidal Carbon Dioxide (PETCO_2_) kept between 35–45 mmHg and BIS values maintained at 40–60 during surgery. Intraoperative analgesia was titrated in real time based on continuous monitoring of vital signs (including blood pressure, heart rate, and mean arterial pressure) and nociceptive response assessment using the Surgical Pleth Index (SPI). SPI values were recorded noninvasively using a GE Healthcare (Finland) patient monitor equipped with a compatible pulse oximetry probe. SPI data were collected at 30-s intervals from the start of surgery until 3 min prior to completion, provided the Bispectral Index remained below 60. An SPI value ranging from 20 to 50 under general anesthesia is indicative of optimal analgesia. A value greater than 50 signals a need for additional analgesia, requiring an increase in the remifentanil dose. Conversely, an SPI value below 20 suggests an overadministration of analgesia, which necessitates a decrease in the infusion rate. Immediately after surgery, PCIA was initiated and the patient transferred to the post-anesthesia care unit (PACU) with the double-lumen endotracheal tube in position. Upon resumption of spontaneous breathing, neostigmine (1 mg) and atropine (0.5 mg) were administered to reverse residual muscle relaxation and the double-lumen endotracheal tube removed.

### 2.5 Study interventions

All patients received ICNB before anesthesia induction. Considering the patient’s anxiety and tension, we routinely administered 0.02–0.03 mg/kg of midazolam for sedation before performing the block. Patients were positioned in a lateral decubitus position with the affected side up and the neck slightly flexed to increase the posterior intercostal space. A linear high-frequency ultrasound probe (frequency: 7–13 MHz) was used to identify the ribs and pleura. A 22-gauge, 80-mm nerve block needle was advanced in-plane under real-time ultrasound guidance. A line was drawn parallel to the spine, 8 cm lateral to the spinal midline, and a skin wheal was raised at the target rib level. The needle was advanced along the inferior border of the rib until it passed the lower edge by approximately 0.2–0.3 cm. Patients were then instructed to hold their breath to minimize pleural movement. After negative aspiration for blood and air, a total of 25 mL of 0.375% ropivacaine was injected to block levels T2 to T6 (5 mL per level). Following negative aspiration, the local anesthetic was injected slowly under continuous ultrasound visualization, with intermittent aspiration to ensure safety. The concentration and volume of local anesthetic were individualized based on the patient’s body weight and specific anesthesia-related risk factors (e.g., mild hepatic or renal dysfunction), following a standardized institutional protocol to optimize safety while maintaining analgesic efficacy, and the total dose did not exceed the maximum recommended safe dose of ropivacaine. The block was performed approximately 20 min before anesthesia induction to ensure adequate local anesthetic spread and onset. Block success was defined as ultrasound visualization of local anesthetic spread along the inferior border of the rib accompanied by pleural displacement.

The PCIA was administered immediately after surgery. For Group C (control group): sufentanil (0.03 μg/kg/h) + tropisetron (10 mg) were diluted in normal saline to a total volume of 100 mL and the infusion performed at a rate of 2 mL/h. A single press provided an additional dose of 1 mL, with a lockout time of 15 min, and continuous infusion was maintained for 48 h postoperatively. The same procedure was conducted for the Group K1 (low-dose esketamine group), which comprised of esketamine (0.015 mg/kg/h) + tropisetron (10 mg) diluted in normal saline to a total volume of 100 mL. Similar procedures for groups K1 and C were followed for Group K2 (moderate-dose esketamine group) which used esketamine (0.03 mg/kg/h) + tropisetron (10 mg) diluted in normal saline to a total volume of 100 mL. In addition to intravenous patient-controlled analgesia (IV-PCA), if the patient’s resting NRS pain score was ≥4, regardless of whether two consecutive PCIA demands had been made, the patient received an intravenous injection of flurbiprofen axetil (50 mg per dose), with a maximum daily dose not exceeding 200 mg.

### 2.6 Primary outcome

The primary outcome involved the assessment of resting and dynamic NRS pain scores at 2, 4, 24, 48, and 72 h postoperatively. After the patient was returned to the ward, trained anesthesiologists or medical staff explained the meaning of the scale to the patients and instructed them to mark their pain level on the scale based on their pain experience. The NRS pain scale (0: no pain, 1–3: mild pain, 4–6: moderate pain, 7–10: severe pain) exhibits reliability and produce valid results (Karcioglu et al., 2018).

### 2.7 Secondary outcomes

(1) postoperative pain assessment, encompassing the incidence of severe pain within 0–24 h and 48–72 h, rescue analgesia rate, PCIA device first press time (hr), and Total dose of flurbiprofen axetil (mg); (2) incidence of postoperative adverse events, including neurological symptoms (dizziness, headache, drowsiness, excessive sedation, dissociation, hallucinations, vivid dreams), dermatological symptoms (pruritus), oculomotor symptoms (nystagmus), gastrointestinal symptoms (nausea and vomiting), and respiratory complications; (3) intraoperative parameters (duration of surgery and anesthesia, blood loss, urine output, volumes of crystalloid and colloid infusion, lobe + lymphadenectomy and direction of surgery); and (4) postoperative recovery (hospital stay duration).

### 2.8 Sample size

The required sample size was determined *a priori* using PASS 15.0 software (NCSS, LLC), based on the primary outcome of the resting NRS score at 24 h postoperatively. Pilot data (30 patients per group) showed mean scores of 4.46 ± 1.05 in the control group and 4.00 ± 0.85 in the 0.03 mg/kg/h esketamine group, yielding a pooled standard deviation (Sp) of 0.96. For a mean difference of 0.46 points (effect size d = 0.48), a two-sample t-test (assuming equal variance) with a two-tailed α of 0.05% and 90% power (1-β = 0.90) required at least 97 participants per group. To account for the three-group design and an estimated 20% dropout rate, the total planned sample size was increased to 360, aiming to enroll 120 patients in each group.

### 2.9 Data precision and variability

In the final analysis, the primary outcome analysis (per-protocol set, n = 120 per group) showed that the NRS scores were 4.98 ± 0.82 in the control group and 3.47 ± 0.65 in the intervention group. The observed absolute between-group difference was 1.51 points, with a pooled standard deviation (Sp) of 0.74, corresponding to a standardized effect size (Cohen’s d) as high as 2.04. This effect size far exceeded the pre-estimated value (d = 0.48) upon which the sample size calculation was based. This confirms that the pre-specified sample size in this study was sufficient and robust for detecting the substantial clinical difference that actually existed.

To address potential concerns about data variability, we calculated the standard deviations and coefficients of variation (CV) for each group. In Supplementary Table S1, Groups C and K1 demonstrated moderate variability (CV range: 8.08%–16.47% and 12.92%–17.66%, respectively), which underscores the precision of our estimates. The higher CVs observed in Group K2 (18.73%–34.66%) indicate a greater degree of dispersion, potentially reflecting differential treatment responses. Similarly, in Supplementary Table S2, Groups C and K1 exhibited low CVs (CV range: 4.09%–10.44% and 3.53%–10.87%, respectively), signifying high data consistency. In contrast, the higher CVs in Group K2 (3.33%–18.58%) suggest increased variability, which may also be attributed to varying treatment responses. This relatively large sample size, along with strict control of measurement errors, improved the precision of effect size estimates and may explain the narrow confidence intervals in Tables 1, 2.

**TABLE 1:** The demographic and clinical characteristics of participants.

Characteristics	Group C (n = 118)	Group K1 (n = 120)	Group K2 (n = 119)	P value
Age (years)	52.7 ± 13.5	55 ± 14.0	56.5 ± 15.1	0.989
Sex (male),n (%)	59 (50.0%)	66 (55.0%)	67 (55.9%)	0.511
Height (cm)	159 ± 7.0	159 ± 7.1	159 ± 7.9	0.523
Weight (kg)	64.3 ± 2.1	66.3 ± 2.5	67.2 ± 2.8	0.752
ASA physical status classification				
I	30 (25.6%)	27 (22.2%)	27 (22.7%)	0.338
II	76 (63.9%)	80 (67.0%)	80 (66.6%)	0.229
III	12 (10.5%)	13 (10.8%)	12 (10.7%)	0.568
Comorbidities				
Diabetes,n (%)	14 (11.9%)	16 (13.3%)	13 (11.2%)	0.532
Hypertension,n (%)	21 (18.1%)	22 (19.1%)	24 (20.1%)	0.852
Coronary heart disease, n (%)	8 (6.5%)	7 (5.9%)	6 (5.3%)	0.521
Cerebral infarction	4 (3.2%)	3 (2.7%)	2 (2.3%)	0.329
Preoperative examination				
Hb (g/dL))	13.4 ± 1.3	13.2 ± 1.2	13.0 ± 1.0	0.245
WBC(×10^9^/L)	6.3 ± 2.6	6.4 ± 2.7	6.5 ± 2.8	0.523
NE (×10^9^/L)	3.9 ± 2.2	4.1 ± 2.1	4.3 ± 1.9	0.348
LYM(×10^9^/L)	2.1 ± 1.1	2.2 ± 1.3	2.1 ± 1.5	0.980
MMSE scores	24.3 ± 2.2	24.9 ± 2.1	25.2 ± 2.0	0.785
Preoperative pain history, n (%)	7 (5.4%)	5 (3.9%)	5 (4.2%)	0.653
Smoking history,n (%)	42 (35.5%)	40 (32.8%)	41 (34.2%)	0.594
Duration of operation (min)	145.1 ± 13.9	143.5 ± 13.8	142 ± 13.4	0.257
Duration of anesthesia (min)	157.4 ± 14.0	153.3 ± 15.2	152.7 ± 15.3	0.564
Bleeding loss (mL)	231.1 ± 163.3	239.3 ± 157.4	240.3 ± 158.3	0.554
Urine volume (mL)	542.9 ± 192.4	537.7 ± 190.2	530.6 ± 187.9	0.664
Crystalloid infusion volume (mL)	867.2 ± 128.7	868.2 ± 142.7	870.2 ± 144.9	0.549
Colloidal fluid infusion volume (mL)	370.1 ± 85.5	372.1 ± 81.9	380.1 ± 79.9	0.785
UniVATS	89 (75.1%)	88 (73.3%)	91 (76.8%)	0.698
Lobe + lymphadenectomy	66 (56.3%)	64 (53.4%)	62 (52.1%)	0.875
Direction of surgery,n (%)				
Right	62 (52.7%)	59 (49.6%)	58 (48.5%)	0.563
Left	56 (47.3%)	61 (50.4%)	62 (51.5%)	0.612

Values are presented as mean ± SD, or counts and percentage (%). Abbreviations: ASA, american society of anesthesiologists physiological status; SD, standard deviation; Hb, Hemoglobin; WBC: leukocyte; NE, neutrophilic granulocyte; LYM, lymphocyte; MMSE, mini-mental state examination; UniVATS, uniportal video-assisted thoracoscopic surgery. Fisher’s exact test was applied for any categorical variables with an expected cell count of less than 5.

**TABLE 2:** Acute postoperative pain at rest.

​	Mean ± SD			Group C vs. Group K1		Group C vs. Group K2		Group K1 vs. Group K2	
Time point	Group C (n = 118)	Group k1 (n = 120)	Group k2 (n = 119)	Mean difference (95% CI)	P value	Mean difference (95% CI)	P value	Mean difference (95% CI)	P value
2h	4.21 ± 0.34	4.32 ± 0.57	2.45 ± 0.52	0.11 (−0.04–0.26)	0.14	−1.76 (−1.91to −1.61)	<0.01	−1.87 (−2.02 to −1.72)	<0.01
4h	4.58 ± 0.70	4.49 ± 0.58	2.51 ± 0.87	−0.09 (−0.31 to 0.13)	0.61	−2.07 (−2.29 to −1.84)	<0.01	−1.98 (−2.20 to −1.76)	<0.01
24h	4.98 ± 0.82	4.87 ± 0.86	3.47 ± 0.65	−0.11 (−0.34 to 0.12)	0.52	−1.51 (−1.74 to −1.27)	<0.01	−1.40 (−1.63 to −1.16)	<0.01
48h	4.29 ± 0.53	4.19 ± 0.74	2.61 ± 0.51	−0.10 (−0.28 to 0.08)	0.41	−1.68 (−1.86 to −1.50)	<0.01	−1.58 (−1.76 to −1.39)	<0.01
72h	3.12 ± 0.37	3.09 ± 0.41	2.45 ± 0.51	−0.03 (−0.16 to 0.10)	0.86	−0.67 (−0.89 to −0.53)	<0.01	−0.64 (−0.77 to −0.50)	<0.01

Data are mean ± SD., Based on the significant interaction from Repeated Measures ANOVA, post-hoc Bonferroni tests were performed at each time point (mean difference with 95% CI). NRS, Numerical Rating Scale.

In addition, we clarified the assumptions underlying the sample size calculation. The variance estimates were based on pilot data, and homogeneity of variance was confirmed using Levene’s test. The narrow confidence intervals likely reflect reduced inter-individual variability due to consistent surgical, anesthetic, and postoperative protocols, which strengthened the internal validity of the study.

### 2.10 Statistical analysis

Statistical analyses were performed using [IBM SPSS Statistics, version 25.0]. Continuous data conforming to a normal distribution are presented as mean ± standard deviation and compared among groups using one-way analysis of variance (ANOVA). Non-normally distributed continuous data are expressed as median (interquartile range) and compared using the Kruskal–Wallis H test. Categorical data are presented as number (percentage) and analyzed using the chi-square test; Fisher’s exact test was applied when any expected cell count was less than 5. For repeatedly measured pain scores, repeated-measures ANOVA was employed. Mauchly’s test of sphericity was conducted, and if the assumption was violated, the Greenhouse–Geisser correction was applied. Post-hoc pairwise comparisons were adjusted using the Bonferroni method, with results reported as mean difference and 95% confidence interval. All tests were two-tailed, and a P value <0.05 was considered statistically significant.

## 3 Results

### 3.1 Study flow and patient characteristics

**FIGURE 1:**
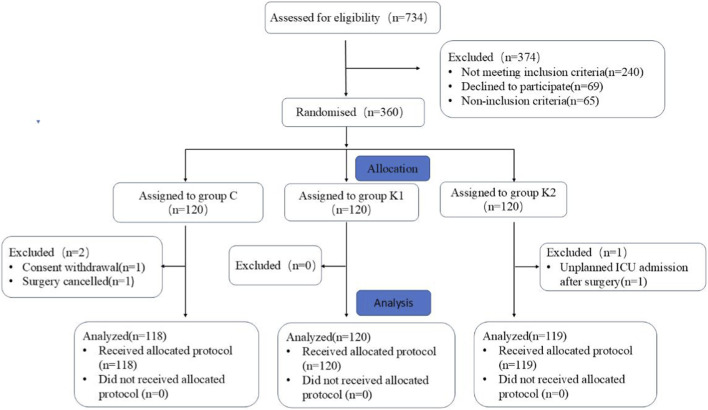
Flow diagram of the study.

A total of 734 patients were screened from October 2021 to July 2023. Among these, 374 patients were excluded based on the exclusion criteria, 69 patients declined to participate, and 65 patients were excluded for various reasons. Ultimately, 360 patients were randomized. In the C group, one patient withdrew their consent after randomization and one patient canceled their scheduled surgery after randomization. In the K2 group, one patient was non-electively transferred to the ICU after surgery. After these changes, final analysis comprised a total of 118 patients from the C group, 120 patients from the K1 group, and 119 patients from the K2 group final analysis (Figure 1). There were no significant differences in the baseline characteristics (demographics, ASA classification, preoperative physical status, Preoperative pain history and smoking history) and surgical data (operative time, anesthesia time, blood loss, urine output, and fluid infusion volume, Lobe + lymphadenectomy and Direction of surgery) among the groups (Table 1).

### 3.2 Primary outcome

The average pain experienced by the three groups of patients at varying time points under resting and movement conditions are displayed in Table 1, 2, respectively. For both resting and active conditions, the highest NRS score occurred at 24 h postoperatively. Under resting conditions at 24 h postoperatively, there was a statistically significant difference in the average NRS pain scores between Group C and Group K2 (4.98 ± 0.82 vs. 3.47 ± 0.65, P < 0.01). Similarly, under active conditions at 24 h postoperatively, the average NRS pain scores between Group C and Group K2 also showed a statistically significant difference (6.85 ± 0.28 vs. 4.51 ± 0.15, P < 0.01). Additionally, at 2, 4, 48, and 72 h postoperatively, the NRS pain scores in Group K2 were significantly lower compared to those in Group C in both states (P < 0.01). Similarly, Group K1 and Group K2 exhibited statistically significant differences in NRS scores at each time point postoperatively (P < 0.01). However, there was no statistically significant difference in NRS scores between Group C and Group K1 at any time point postoperatively (P > 0.05).

Repeated measures ANOVA further revealed the patterns of pain scores over time and across groups. Under resting conditions (Table 2), the analysis showed a significant main effect of group (F (2, 354) = 253.5, P < 0.001). A significant main effect of time was also found (F (4, 1770) = 101.7, P < 0.001). Moreover, a significant group × time interaction was observed (F (8, 1770) = 52.4, P < 0.001), indicating that the trends of pain changes over time differed among the groups.

Under active conditions (Table 3), significant main effects of group (F (2, 1770) = 2,750.16, P < 0.001) and time (F (4, 1770) = 1,132.06, P < 0.001), as well as a significant group × time interaction (F (8, 1770) = 49.38, P < 0.001), were also observed. Line graphs showing the changes in pain scores over time can be found in Supplementary Material Supplementary Figure S1.

**TABLE 3:** Acute postoperative pain on movement.

​	Mean ± SD			Group C vs. Group K1		Group C vs. Group K2		Group K1 vs. Group K2	
Time point	Group C (n = 118)	Group k1 (n = 120)	Group k2 (n = 119)	Mean difference (95% CI)	P value	Mean difference (95% CI)	P value	Mean difference (95% CI)	P value
2h	6.13 ± 0.45	6.14 ± 0.49	4.19 ± 0.53	0.01 (−0.14–0.16)	0.99	−1.94 (−2.09 to −1.79)	<0.01	−1.95 (−2.10to −1.80)	<0.01
4h	6.42 ± 0.67	6.22 ± 0.63	4.26 ± 0.69	−0.20 (−0.40 to 0.01)	0.06	−2.16 (−2.36 to −1.96)	<0.01	−1.96 (−2.16 to −1.90)	<0.01
24h	6.85 ± 0.28	6.79 ± 0.24	4.51 ± 0.15	−0.06 (−0.13 to 0.01)	0.11	−2.34 (−2.41 to −2.27)	<0.01	−2.28 (-2.35to −2.21)	<0.01
48h	5.54 ± 0.54	5.43 ± 0.59	3.39 ± 0.63	−0.11 (−0.29 to 0.07)	0.32	−2.15 (−2.33 to −1.97)	<0.01	−2.04 (−2.22 to −1.86)	<0.01
72h	4.13 ± 0.41	4.02 ± 0.38	3.18 ± 0.45	−0.11 (-0.24 to 0.02)	0.10	−0.95 (−1.07 to −0.82)	<0.01	−0.84 (−0.97 to −0.71)	<0.01

Data are mean ± SD., Based on the significant interaction from Repeated Measures ANOVA, post-hoc Bonferroni tests were performed at each time point (mean difference with 95% CI). NRS, Numerical Rating Scale.

### 3.3 Secondary outcomes

#### 3.3.1 Incidence of APP within 48 h after surgery

**FIGURE 2:**
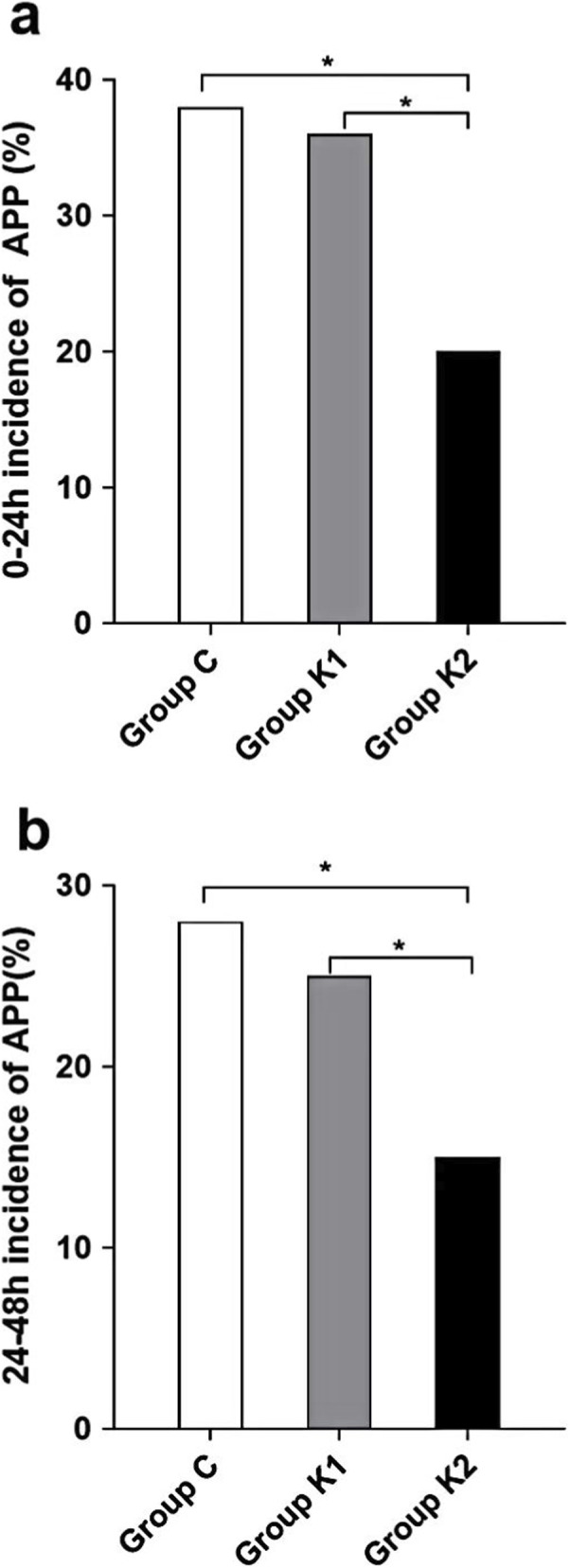
Incidence of acute pain during 0‐24 **(a)** and 24‐48 h **(b)** after surgery. *p < 0.05.

Postoperative NRS ≥4 was defined as an inadequate APP control. Within 24 h postoperatively (Figure 2a), among 118 patients in Group C, 45 (38%) experienced APP; in Group K1, 43 (36%) out of 120 patients experienced APP; in Group K2, 23 (20%) out of 119 patients experienced APP. The incidence of APP in Group C and Group K1 was significantly higher compared to that in Group K2 (P < 0.05). Between 24–48 h postoperatively (Figure 2b), among 118 patients in Group C, 33 (28%) experienced APP; in Group K1, 30 (25%) out of 120 patients experienced APP; in Group K2, 17 (15%) out of 119 patients experienced APP. The incidence of severe pain in Group C and Group K1 was significantly higher compared to that in Group K2 (P < 0.05).

#### 3.3.2 Occurrence of postoperative adverse reactions

Within 72 h postoperatively, the incidence of postoperative nausea and vomiting in Group K2 was significantly lower compared to that in Group C and Group K1 (P < 0.01). Postoperative nausea and vomiting occurred in 41 cases (34.88%) in Group C, 14 cases (11.8%) in Group K1, and 12 cases (10.1%) in Group K2. There were no significant differences in the incidence of postoperative dizziness, headache, pruritus, drowsiness, nystagmus, dissociation, nausea/vomiting, hallucinations, vivid dreams, excessive sedation and respiratory complications among the three groups (P > 0.05) (Table 4).

**TABLE 4:** Comparison of postoperative adverse reactions.

Adverse events	Group C (n = 118)	Group K1 (n = 120)	Group K2 (n = 119)	P value
Dizziness	8 (6.97)	5 (4.44)	3 (2.54)	0.156
Headache	7 (6.50)	4 (3.09)	2 (1.09)	0.158
Pruritus	0 (0.00)	0 (0.00)	0 (0.00)	0.213
Drowsiness	7 (6.63)	2 (2.22)	3 (2.15)	0.098
Nystagmus	2 (2.32)	0 (0.00)	0 (0.00)	0.198
Separation sensation	0 (0.00)	3 (2.42)	5 (4.54)	0.125
Nausea and Vomit	41 (34.88)	14 (11.86)	12 (10.08)	<0.01
Hallucinations	0 (0.00)	0 (0.00)	0 (0.00)	0.256
Vivid dreams	0 (0.00)	0 (0.00)	0 (0.00)	0.268
Excessive sedation	0 (0.00)	0 (0.00)	0 (0.00)	0.345
Respiratory complications	2 (1.69)	1 (0.83)	0 (0.00)	0.082

Data are presented as n (%). Group C was given intravenous controlled analgesia using sufentanil 0.03 ug/kg/h in PCIA, Group K1 was administered with PCIA, at low dosages of esketamine 0.015 mg/kg/h, and Group K2 was administered with PCIA, at middle dosages of esketamine 0.03 mg/kg/h. Fisher’s exact test was applied for any categorical variables with an expected cell count of less than 5. Respiratory complications include: Atelectasis, Pleural Effusion, Bronchospasm, and Hypoxemia. Excessive sedation was defined as a Ramsay Sedation Scale score of greater than 4.

#### 3.3.3 Comparison of postoperative analgesic efficacy and medication differences among intervention groups

This table compares key postoperative analgesic indicators among Group C, K1, and K2. The data show that, compared to Group C and K1, Group K2 had a significantly later first press time for PCIA, significantly lower total consumption of flurbiprofen axetil, and a significantly lower proportion of patients requiring rescue analgesia within both 0–24 h and 24–48 h after surgery. However, there was no statistically significant difference in the average hospitalization duration among the three groups. The results suggest that the intervention protocol in Group K2 may provide superior and longer-lasting postoperative analgesia, reducing the need for analgesic medications, but without shortening hospital stay (Table 5).

**TABLE 5:** Comparison of postoperative analgesic efficacy and medication consumption.

Outcomes	Group C (n = 118)	Group K1 (n = 120)	Group K2 (n = 119)	F/X2	P value
PCIA device first press time (hr)	2.3 ± 1.6	2.5 ± 1.2	3.4 ± 1.7	17.78	p < 0.01
Total dose of flurbiprofen axetil (mg)	158.9 ± 33.2	152.6 ± 44.1	115.0 ± 23.1	55.99	p < 0.01
Rescue analgesia,n (%)					
0–24h	45 (38.13)	43 (35.83)	24 (20.16)	10.56	p < 0.01
24–48h	34 (28.81)	30 (25.00)	18 (15.12)	6.69	p < 0.05
Hospitalization duration (d)	7.5 ± 2.9	7.2 ± 2.3	7.1 ± 3.1	0.66	P = 0.517

Data are presented as mean ± standard deviation or number (%). A p-value <0.05 was considered statistically significant. Specific test statistics (F or X^2^) are provided in the table.

## 4 Discussion

The results of this study validate that the use of esketamine as an alternative for opioid medications in postoperative PCIA combined with preoperative ICNB effectively relieve APP in patients undergoing VATS lobectomy. The core of this analgesic strategy lies in achieving an opioid-sparing effect. By reducing opioid exposure, it directly mitigates the associated dose-dependent adverse reactions, such as respiratory depression, excessive sedation, and gastrointestinal dysfunction. Thereby, it enhances perioperative patient safety and comfort, and lays the foundation for promoting early mobilization, accelerating functional recovery, and shortening hospital stay. This approach aligns closely with the principles of ERAS (Hu et al., 2025).

Esketamine plays a pivotal role in this strategy. Its analgesic effect stems from a multi-target synergistic mechanism: as an NMDA receptor antagonist, it effectively blocks central sensitization and hyperalgesia (Sarmah et al., 2022); by activating μ- and δ-opioid receptors, it not only modulates pain signal transduction but may also ameliorate opioid tolerance (Mizobuchi et al., 2022); furthermore, it promotes the release of norepinephrine and serotonin, thereby augmenting the function of descending pain inhibitory pathways (Wang et al., 2025). A meta-analysis indicated that the efficacy and adverse effects of esketamine are influenced by the timing of administration, the method of application, and the dosage. Specifically, compared with intraoperative administration alone, the combination of intraoperative and postoperative administration significantly reduces resting pain scores at 24 h postoperatively (Wang et al., 2021). However, Brinck et al. pointed out that the analgesic effect of intravenously administered S-ketamine during lumbar fusion surgery is transient and independent of the dosage (Brinck et al., 2021). Additionally, a study has found that compared with a single bolus injection, continuous low-dose infusion of esketamine over 24–48 h provides superior analgesic efficacy for postoperative APP and reduces the incidence of postoperative complications (Zhou et al., 2023). Therefore, optimizing the dosage of esketamine has always been a challenge for clinicians.

There are studies indicate that incorporating esketamine (0.two to one mg/kg) to postoperative PCIA significantly enhance analgesic efficacy, inhibit inflammatory responses, and improve sleep quality and early postoperative cognitive function in elderly patients (Kohtala, 2021). In a randomized, double-blind, controlled trial, researchers recommend the incorporation of esketamine to opioid-based PCIA for thoracic surgery patients, at dose range of 0.02–0.04 mg/kg/h. However, this study also note that a dose of 0.04 mg/kg/h of esketamine for postoperative intravenous pain relief lead to stronger sedative effects, which exhibits potential clinical safety risks (Zhang et al., 2024). This could potentially compromise its advantages in facilitating accelerated postoperative recovery and raise safety concerns. Therefore, the present study aimed to investigate the efficacy of a lower-dose esketamine regimen within a defined safety window (i.e., 0.015–0.03 mg/kg/h), with the objective of establishing a more favorable balance between efficacy and safety. Additionally, a study on open abdominal surgery has demonstrated that S-ketamine administered at a dosage of 0.015 mg/kg/h is as effective as the conventional low-dose regimen in reducing postoperative opioid consumption and hyperalgesia. Moreover, it has been found to decrease the incidence of postoperative delirium (Zhu et al., 2023). In preliminary trials, we found that low-dose esketamine (0.015 mg/kg/h) in PCIA exhibited similar clinical efficacy to sufentanil (0.03 μg/kg/h) in PCIA. Consequently, preoperative ICNB combined with two doses (0.015 and 0.03 mg/kg/h) esketamine analgesia protocol were used in this study.

The results indicate that both at rest and during movement, the NRS scores in the K2 group were lower compared to those in the remaining groups, indicating that the perioperative ICNB combined with 0.03 mg/kg/h esketamine in PCIA exhibits superior analgesic efficacy compared to the remaining groups. Further analysis revealed that the incidence of APP within 48 h postoperatively in the C group was 38% while in the K1 group it was 36%, which is consistent with the findings of Bendixen M et al. (Bendixen et al., 2016). Notably, the incidence of APP within 48 h postoperatively was approximately 20% in the K2 group, representing a 34% relative reduction compared to the C group. Generally, a reduction in pain intensity by 30% is considered clinically significant (Cepeda et al., 2003). In contrast, the incidence of APP in the K1 group (36%) was similar to that in Group C. This inadequate analgesic efficacy is likely attributable to an insufficient dosage. Current evidence indicates that the analgesic effect of esketamine is closely associated with its plasma concentration, with an effective analgesic threshold generally considered to be ≥ 0.05 mg/L (this value is derived from half of the recommended clinical dose of ketamine) (Miller’s basics of anesthesia, 2024). The dosage used in the K1 group (0.015 mg/kg/h via continuous infusion) may have resulted in a steady-state plasma concentration below this threshold, leading to suboptimal analgesia. This finding also suggests that in entirely opioid-sparing analgesic regimens, dose optimization of non-opioid agents is crucial. Conversely, a classic multimodal approach combining low-dose opioids with esketamine may represent another viable option for balancing analgesic adequacy and safety, warranting direct comparison in future studies.

It is well-established that inadequately managed APP is a key risk factor for the development of chronic postoperative pain (CPP) (Marshall and McLaughlin, 2020). Our study focused on the acute postoperative period (within 72 h), and did not assess long-term outcomes such as the incidence CPP. A meta-analysis indicates that the analgesic effect of esketamine is limited (Wang et al., 2021). Moreover, based on the pharmacokinetic calculations of esketamine, after continuous infusion at 0.03 mg/kg/h for 48 h postoperatively, the drug can be nearly completely metabolized and eliminated from the body approximately 24 h after stopping the infusion (Kamp et al., 2020). Nevertheless, we acknowledge the absence of long-term follow-up as a limitation of this study.

Additionally, the incidence of PONV was approximately 34.88% in Group C (sufentanil-based), which was significantly reduced to 10% in Group K2 (0.03 mg/kg/h esketamine). Despite their potent analgesic effects, opioids remain a key component of postoperative pain management; however, their use has been established as an independent risk factor for PONV in numerous studies. Mechanistically, opioids induce nausea and vomiting not only by activating μ-opioid receptors, which reduces gastrointestinal secretion and motility, but also by directly stimulating the chemoreceptor trigger zone in the medulla oblongata (Yates et al., 1998; Takagi et al., 2024). Therefore, replacing opioids with esketamine in postoperative analgesia, combined with preoperative intercostal nerve block (ICNB), significantly reduces the incidence of PONV and may consequently attenuate the occurrence of acute postoperative pain (APP) in thoracic surgery patients. This “anti-PONV effect” of esketamine essentially represents a collateral benefit arising from its opioid-sparing analgesic strategy (Feng et al., 2024). Within a multimodal analgesic regimen, esketamine not only provides effective analgesia but also avoids the direct stimulation of gastrointestinal function and central vomiting pathways typically induced by opioids, thereby markedly lowering the risk of PONV. It is noteworthy that the esketamine doses used in this study were relatively low, and no related psychiatric symptoms were observed in either the K1 or K2 groups. This finding aligns with recent literature indicating that subanesthetic doses of esketamine can achieve effective analgesia while minimizing dissociative symptoms and psychiatric side effects (Johnston et al., 2024).

In addition to its analgesic effects, esketamine exhibits sedative properties. ^18^ The mechanism underlying its sedative effects is associated with the non-competitive inhibition induced by its binding to NMDA receptors (Vyklicky et al., 2014). Recent research show that esketamine alleviate postoperative anxiety, depression-like symptoms, and cognitive dysfunction in mice by inhibiting M1 polarization of microglial cells and inflammatory responses, while improving the BDNF-TrkB signaling pathway both *in vitro* and *in vivo* (Fedgchin et al., 2019). Additionally, an increasing number of clinical studies have found that intraoperative administration of sub-anesthetic doses of esketamine alleviate postoperative anxiety, depression, and certain levels of pain (Hannon et al., 2023; McIntyre et al., 2021; Tully et al., 2022), as well as improving postoperative sleep quality (Richards et al., 2025; Tsze et al., 2012). This study also found that although the incidence of PONV was significantly reduced in both the K1 and K2 groups compared to the C group, the occurrence of APP in the K1 group did not significantly decrease. Therefore, the results of this study suggest that preoperative ICNB combined with a postoperative analgesic regimen of 0.03 mg/kg/h esketamine in PCIA should be recommended.

## 5 Limitations

This study has several limitations. First, Although the sample size of this study was sufficient for testing the primary hypothesis, it remains limited for assessing rare adverse events or conducting certain subgroup analyses. Second, the surgical approach may have influenced the results. More than 75% of the patients in this study underwent uniportal video-assisted thoracic surgery, which is associated with relatively mild postoperative pain compared to multiportal approaches. Therefore, caution is warranted when extrapolating the findings to patients undergoing biportal or triportal procedures. Although subgroup analysis was attempted for multiportal cases, the small sample size in these subgroups limited the statistical power and precluded definitive conclusions. Third, neuromuscular blockade management relied on clinical assessment rather than quantitative train-of-four monitoring. The incorporation of objective monitoring in future studies would improve the accuracy of recovery evaluation. Fourth, the absence of esketamine plasma concentration monitoring prevented a clear determination of whether the inferior analgesic efficacy in the low-dose esketamine group was attributable to subtherapeutic drug levels and hindered systematic pharmacokinetic-pharmacodynamic analysis. Fifth, the relatively high incidence of acute postoperative pain in the low-dose esketamine group suggests that the analgesic efficacy of this regimen alone remains uncertain. Moreover, the lack of a control group receiving low-dose opioids combined with esketamine precluded a direct comparison with the mainstream opioid-sparing multimodal analgesia strategy. Finally, the absence of long-term follow-up limited the assessment of the potential effects of esketamine on chronic pain and postoperative cognitive function. Future studies should address these limitations by incorporating extended follow-up, drug concentration monitoring, and more diverse control designs.

## 6 Conclusion

A dose of 0.03 mg/kg/h esketamine in PCIA combined with preoperative ICNB significantly alleviates APP in patients undergoing thoracoscopic lobectomy, reducing resting pain scores by approximately 30% at 24 h compared to sufentanil-based analgesia.

## Data Availability

The raw data supporting the conclusions of this article will be made available by the authors, without undue reservation, to any qualified researcher. No URL link is provided as the data are not deposited in a public repository; interested parties may contact the corresponding author directly.
