# Supplementary material for: Esketamine-based PCIA combined with intercostal nerve block for acute pain after lobectomy: a randomized controlled trial
Source: Front Pharmacol. 2026 Mar 4;17:1746121. doi: 10.3389/fphar.2026.1746121 (PMC12996927; doi:10.3389/fphar.2026.1746121)

Table S1 Results of the coefficient of variation in the resting state

| Times | Group C (n=118) | Group K1  (n=120) | Group K2  (n=119) |
| --- | --- | --- | --- |
| 2h | **8.08%** | **13.19%** | **21.22%** |
| 4h | **15.28%** | **12.92%** | **34.66%** |
| 24h | **16.47%** | **17.66%** | **18.73%** |
| 48h | **12.35%** | **17.66%** | **19.54%** |
| 72h | **11.86%** | **13.27%** | **20.82%** |

TableS2 Results of the coefficient of variation in the movement state

| Times | Group C (n=118) | Group K1  (n=120) | Group K2  (n=119) |
| --- | --- | --- | --- |
| 2h | **7.34%** | **7.98%** | **12.65%** |
| 4h | **10.44%** | **10.13%** | **16.20%** |
| 24h | **4.09%** | **3.53%** | **3.33%** |
| 48h | **9.75%** | **10.87%** | **18.58%** |
| 72h | **9.93%** | **9.45%** | **14.15%** |
|  |  |  |  |

Figure S1 Visualization of the interaction effect


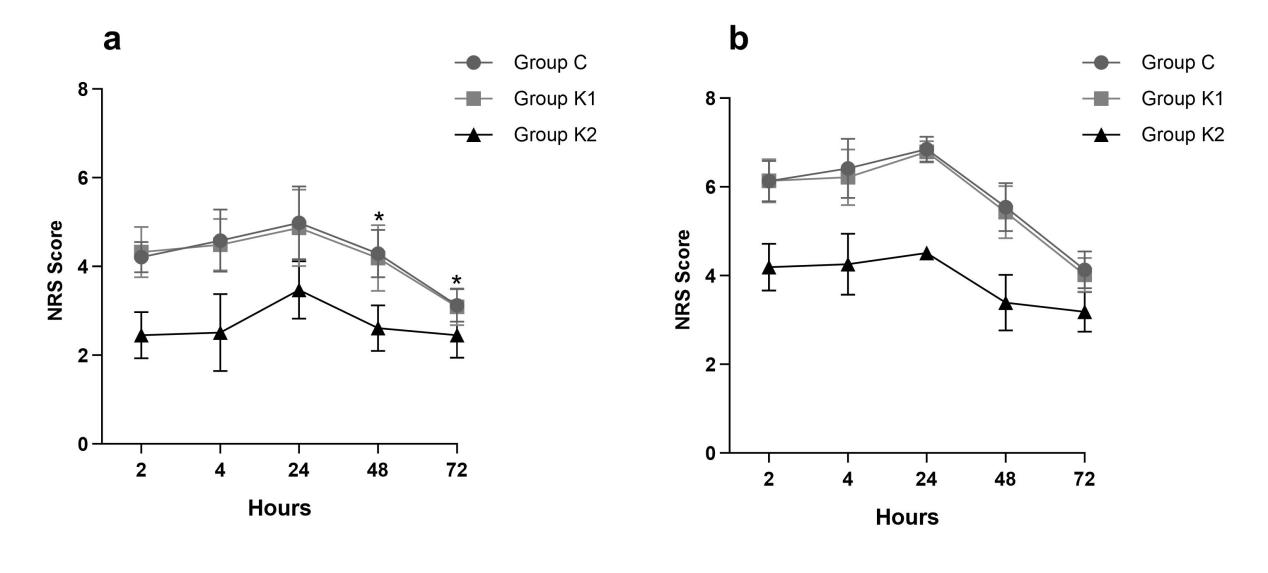

Supplement: Supplementary file 1 [file Supplementaryfile1.docx]
